# Mumps, Cervical Zoster, and Facial Paralysis: Coincidence or Association?

**DOI:** 10.1155/2014/289687

**Published:** 2014-02-06

**Authors:** Kenji Kondo, Kaori Kanaya, Shintaro Baba, Tatsuya Yamasoba

**Affiliations:** Department of Otorhinolaryngology-Head and Neck Surgery, Graduate School of Medicine, The University of Tokyo, 7-3-1 Hongo, Bunkyo-ku, Tokyo 113-8655, Japan

## Abstract

The association of mumps with peripheral facial paralysis has been suggested, but its pathogenesis remains unclear. An 8-year-old girl simultaneously developed left peripheral facial paralysis, ipsilateral cervical herpes zoster, and bilateral mumps sialadenitis. Elevated anti-mumps and anti-varicella zoster virus IgM antibodies in serological testing indicated recent infection of mumps and reactivation of VZV. Molecular studies have provided mounting evidence that the mumps virus dysregulates the host's immune system and enables the virus to proliferate in the infected host cells. This dysregulation of the immune system by mumps virus may have occurred in our patient, enabling the latent VZV infection to reactivate.

## 1. Introduction

A variety of virus infections have been linked to the development of peripheral facial paralysis. These viruses include herpes simplex virus-1 (HSV-1), varicella zoster virus (VZV), Epstein-Barr virus, cytomegalovirus, and mumps virus. Increasing evidence suggests that idiopathic peripheral facial paralysis (Bell's palsy), the most common clinical entity of facial paralysis, is caused by reactivation of HSV-1 [1, 2]. Similarly, it is well recognized that VZV reactivation is responsible for the development of Ramsay Hunt syndrome and zoster sine herpete [3]. However, the association of other viral infections with the pathogenesis of facial paralysis remains largely unclear because of the paucity of reports.

We present here a patient who showed the simultaneous development of left peripheral facial paralysis, ipsilateral cervical herpes zoster, and mumps sialadenitis as evidenced by serological studies. Our case, as well as those in previous reports, raises the question whether the so-called mumps-associated facial paralysis is simply caused by mumps virus infection or the additional reactivation of latent VZV.

## 2. Case Presentation

An 8-year-old Japanese girl was referred to our clinic for the evaluation of a left facial nerve paralysis, left cervical zoster, and swelling of the right submandibular area. She developed cervical zoster and facial paralysis on the first and second day of illness, respectively, and was given prednisolone (1 mg/kg/day) and valaciclovir (1500 mg/day) orally by a pediatrician from the fourth day of illness. She subsequently developed a right submandibular swelling on the eighth day. She did not notice hearing loss or vertigo. She had been vaccinated against mumps at the age of 2 and had no obvious episode of mumps infection previously. Although she had also been vaccinated for chickenpox at the age of 2, she developed chickenpox at the age of 4. Another past medical history was notable for an operation for Fallot's tetralogy at the age of 2.

**Figure 1:**
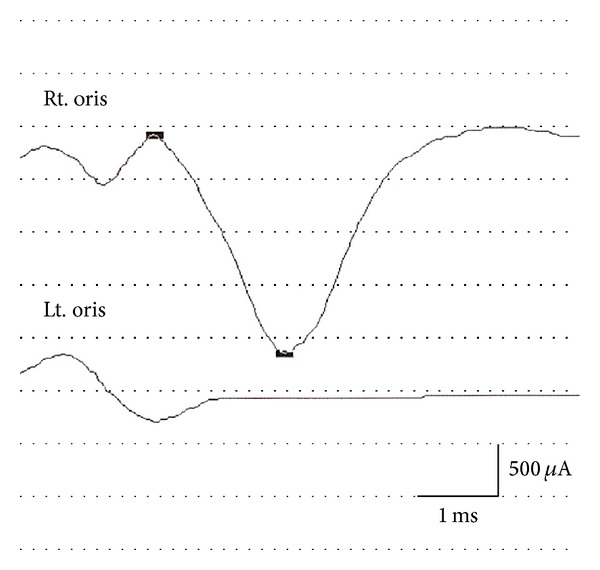
An electroneurograph recorded from the orbicularis oris muscles on the tenth day of illness showing severe denervation of the left facial nerve. Supramaximal stimulation was provided through bipolar surface electrodes placed with the anode just outside the stylomastoid foramen and the cathode in front of the ear lobe.

**Figure 2:**
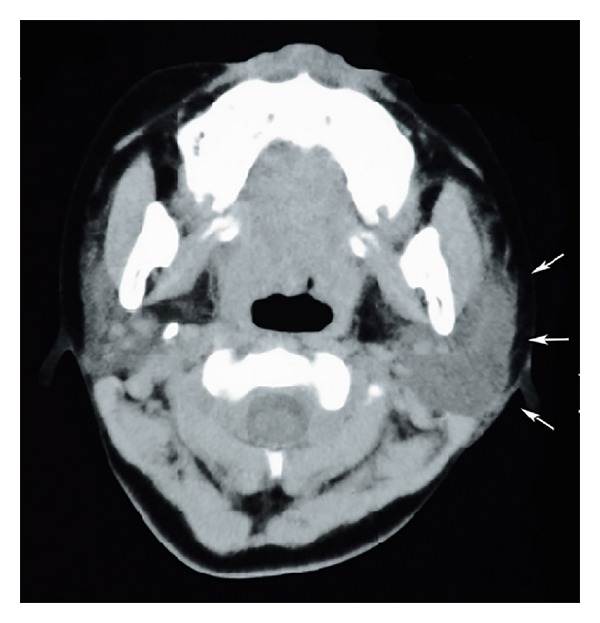
A computed tomography image of the neck reveals the swelling of the left parotid gland (arrows).

**Figure 3:**
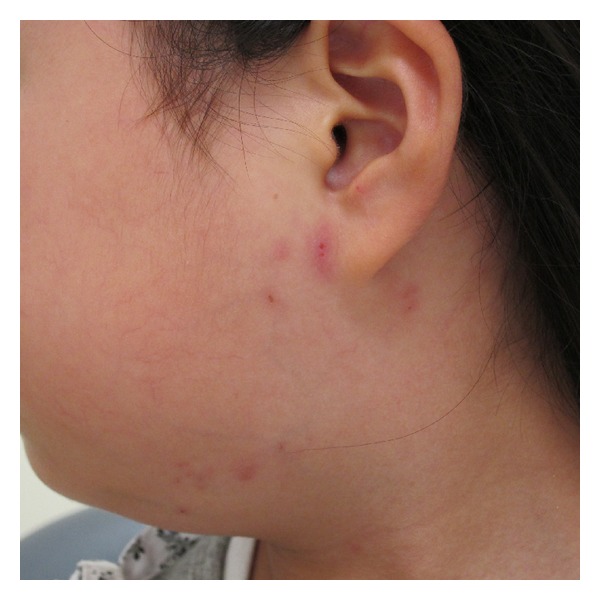
A photograph showing the cervical herpes zoster in the left parotid and cervical area.

At the first visit to our clinic on the tenth day of illness, a physical examination revealed a complete left facial paralysis (House-Brackmann grade VI). Electroneurography of the facial nerve revealed nearly complete denervation of the left facial nerve (Figure 1). Pure tone audiometry showed normal hearing in both ears. Stapedial reflex was absent in the left ear. The patient also had swelling of the parotid glands bilaterally and the right submandibular gland. Computed tomography images of the neck that had been examined in the pediatric department on the eighth day of illness confirmed sialadenitis of the left parotid gland (Figure 2) and right submandibular gland. We also noted herpes zoster in the left parotid area and posterior cervical area corresponding to the C2-3 area (Figure 3). Serologic tests done on the 17th day of illness revealed the elevation of both antimumps and anti-VZV IgM antibodies by an enzyme-linked immunosorbent assay (Table 1).

Her sialadenitis disappeared within 1 week. Her left facial movement also gradually improved to be House-Brackmann grade 2 with slight synkinesis 7 months later. Follow-up serological tests 6 weeks after the onset of illness revealed a decrease in both IgM antimumps and anti-VZV antibodies (Table 1).

## 3. Discussion

To our knowledge, the present report is the first to show simultaneous development of mumps sialadenitis and reactivation of VZV, both of which were evidenced by serological assays, in a case of peripheral facial paralysis. In the present case, cervical zoster and facial paralysis developed six and seven days earlier than the development of sialadenitis, respectively. The electroneurography of the facial nerve revealed nearly complete denervation on the tenth day of illness.

Based on the clinical and laboratory findings, this case may be clinically diagnosed as mumps-associated facial paralysis. In fact, a small number of previous reports have suggested the possible association of mumps viral infection with peripheral facial paralysis [4–6]. However, stapedial reflex in the present case was absent in the left ear, suggesting that the lesion responsible for facial paralysis was not located in the parotid gland but proximal to the root of the stapedius branch, which is commonly observed in cases of Bell's palsy or Ramsay Hunt syndrome. It would be more likely that the facial paralysis was caused by the VZV reactivation. Although the patient did not show hearing loss, or vesicular eruptions in external ear canal, concha, or pimma, which is a dermatome of the geniculate ganglion, a combination of facial paralysis and upper cervical zoster has been recognized as a subgroup of Ramsay Hunt syndrome [7, 8] and speculated to be caused by the simultaneous reactivation of latent VZV in multiple sensory ganglia or the spread of infection through meningeal inflammation to adjacent ganglia [8].

Although it is not clear if the mumps virus infection and VZV reactivation were merely coincidental or were associated in the present case, the time lapse between the two indicates the likelihood of their relation. Assuming that they were actually associated, it is reasonable to speculate that the mumps infection enabled the subsequent reactivation of VZV, since the herpes zoster is a result of reactivation, not the initial infection, of the VZV virus. The latency period of the mumps virus is usually 2-3 weeks, suggesting that mumps infection in our case may have occurred 1-2 weeks before the development of cervical zoster and facial paralysis. Recent molecular studies have provided mounting evidence that the mumps virus may dysregulate the immune system of the host so that the virus can proliferate in the infected host cells [9]. Such a dysregulation of immune system may have occurred in our patient, enabling the latent VZV infection to reactivate. A similar causative association has been suggested in EB virus infection and VZV reactivation [10].

Interestingly, besides the small number of case reports describing the development of peripheral facial paralysis in patients with mumps sialadenitis, the association of trigeminal herpes zoster and mumps parotitis has also been reported [11], as well as an increasing titer of mumps IgM in clinically diagnosed cases of Ramsay Hunt syndrome (without clinical sialadenitis) [12]. With the addition of our case, further speculation may be possible that these cases may represent the incomplete clinical presentation of mumps virus infection and subsequent VZV reactivation.

## 4. Conclusion

The present case suggests that the reactivation of VZV may be associated with “mumps-induced” facial paralysis. It would be beneficial for physicians who manage facial nerve paralysis to keep this possible pathology in mind and to perform serological tests for VZV when encountering the patients with peripheral facial paralysis and mumps sialadenitis, even if they do not show signs of VZV reactivation.

## Figures and Tables

**Table 1:** Results of the serological assays.

Antibody	EIA value at the 17th day	EIA value at the 6th week	Normal range
HSV-IgG	2.1	2.0	<2.0
HSV-IgM	0.77	0.25	<0.8
VZV IgG	848	310	<2.0
VZV IgM	1.38	0.25	<0.8
Mumps IgG	128	128	<2.0
Mumps IgM	3.19	0.98	<0.8
